# Weber–Fechner law in temporal difference learning derived from control as inference

**DOI:** 10.3389/frobt.2025.1649154

**Published:** 2025-09-25

**Authors:** Keiichiro Takahashi, Taisuke Kobayashi, Tomoya Yamanokuchi, Takamitsu Matsubara

**Affiliations:** 1 Division of Information Science, Nara Institute of Science and Technology, Ikoma, Japan; 2 National Institute of Informatics (NII) and The Graduate University for Advanced Studies (SOKENDAI), Tokyo, Japan

**Keywords:** reinforcement learning, temporal difference learning, control as inference, reward–punishment framework, Weber–Fechner law, robot control

## Abstract

This study investigates a novel nonlinear update rule for value and policy functions based on temporal difference (TD) errors in reinforcement learning (RL). The update rule in standard RL states that the TD error is linearly proportional to the degree of updates, treating all rewards equally without any bias. On the other hand, recent biological studies have revealed that there are nonlinearities in the TD error and the degree of updates, biasing policies towards being either optimistic or pessimistic. Such biases in learning due to nonlinearities are expected to be useful and intentionally leftover features in biological learning. Therefore, this research explores a theoretical framework that can leverage the nonlinearity between the degree of the update and TD errors. To this end, we focus on a *control as inference* framework utilized in the previous work, in which the uncomputable nonlinear term needed to be approximately excluded from the derivation of the standard RL. By analyzing it, the Weber–Fechner law (WFL) is found, in which perception (i.e., the degree of updates) in response to a change in stimulus (i.e., TD error) is attenuated as the stimulus intensity (i.e., the value function) increases. To numerically demonstrate the utilities of WFL on RL, we propose a practical implementation using a reward–punishment framework and modify the definition of optimality. Further analysis of this implementation reveals that two utilities can be expected: i) to accelerate escaping from the situations with small rewards and ii) to pursue the minimum punishment as much as possible. We finally investigate and discuss the expected utilities through simulations and robot experiments. As a result, the proposed RL algorithm with WFL shows the expected utilities that accelerate the reward-maximizing startup and continue to suppress punishments during learning.

## 1 Introduction

Reinforcement learning (RL) (Sutton and Barto, 2018) provides robots with policies that allow them to interact in unknown and complex environments, replacing conventional model-based control with it. Temporal difference (TD) learning (Sutton, 1988) is a fundamental methodology in RL. For example, it has been introduced as the basis for proximal policy optimization (PPO) (Schulman et al., 2017) and soft actor-critic (SAC) (Haarnoja et al., 2018), the most famous algorithms in recent years, both of which are implemented on popular RL libraries (Raffin et al., 2021; Huang et al., 2022) and applied to many real robots (Andrychowicz et al., 2020; Wahid et al., 2021; Nematollahi et al., 2022; Kaufmann et al., 2023; Radosavovic et al., 2024). In TD learning, the future value predicted from the current state is compared to that from the state after transition, which is the so-called TD error. The value function for that prediction can be learned by making this TD error 0, and its learning convergence is theoretically supported by the Bellman equation (although some residuals tend to remain in practice). In addition, actor-critic methods often utilize the TD error as the weight of the policy gradient (Sutton et al., 1999) since it indicates the direction of maximizing the future value.

Although TD learning plays an important role in RL theories and algorithms as above, TD learning can explain many biological behaviors. In particular, a strong correlation between TD errors and the amount of dopamine or the firing rate of dopamine neurons, which affects memory and learning in organisms, has been reported (Schultz et al., 1993; O’Doherty et al., 2003; Starkweather and Uchida, 2021), and behavioral learning in organisms is also hypothesized to be based on RL (Dayan and Balleine, 2002; Doya, 2021). Recently, a more detailed investigation of the relationship between TD errors and dopamine has revealed that it is not a simple linear relationship, as suggested by standard TD learning, but is biased and nonlinear (Dabney et al., 2020; Muller et al., 2024). It has also been reported that some of the nonlinearities may stabilize learning performance (Hoxha et al., 2025). In the context of RL theory, nonlinearly transformed TD learning has been proposed to obtain risk-sensitive behavior (Shen et al., 2014; Noorani et al., 2023) and robustness to outliers (Sugiyama et al., 2009; Cayci and Eryilmaz, 2024). The above studies suggest that the implicit biases introduced by nonlinearities would be effective both theoretically and biologically. In other words, discovering new nonlinearities theoretically or experimentally and understanding their utilities have both an engineering value, such as robot control, and a biological value, such as modeling the principles of behavioral learning in organisms. The aim of this study is to discover new nonlinearities theoretically and reveal their functions experimentally, standing on a constructivist approach using robots (Kuniyoshi et al., 2007).

Moreover, our previous study has found that conventional TD learning can be approximately derived using *control as inference* (Levine, 2018), given appropriate definitions of optimality and divergence (Kobayashi, 2022b). At the same time, it also revealed that updating the value and policy functions according to TD errors becomes optimistic by modifying the definition of the divergence. In a subsequent study, it was additionally found that modifying the definition of optimality leads to pessimistic updates (Kobayashi, 2024b). Thus, RL based on *control as inference* has the capacity to capture various nonlinearities due to the generality of the optimization problems it addresses. This study also follows the new derivation of TD learning in these previous studies to find/investigate the novel nonlinearity undiscovered so far.

In particular, we focus on the fact that an approximation was necessary to derive the conventional TD learning from control as inference with linearity between the TD errors and the degrees of updating. This approximation was generally unavoidable to eliminate an unknown variable and allow numerical computation. However, as the term excluded by the approximation is nonlinear, it should be worth analyzing its utilities as the first contribution of this study. To numerically evaluate the utilities, we propose a novel biologically plausible algorithm that combines a reward–punishment framework (Kobayashi et al., 2019; Wang et al., 2021) with a modified definition of optimality (Kobayashi, 2024b), making the nonlinear term computable in any task covered by RL. In this study, biological plausibility is defined as the presence of nonlinearities in organisms within contexts that are beyond learning.

As a result, we show analytically that the nonlinear term, which has been previously excluded, gives rise to the *Weber*–*Fechner law* (WFL), a well-known biologically plausible characteristic (Scheler, 2017; Portugal and Svaiter, 2011; Nutter and Esker, 2006; Binhi, 2023). In particular, the degree of update of the value and policy functions corresponding to the intensity of perception is logarithmically affected by the scale of the value function, which is the base stimulus: with the small scale, the update is sensitive to even a small TD error; with the large scale, only a large TD error allows the update enough. This WFL is dominant when the optimality is highly uncertain, while the conventional linear behavior is found when the optimality becomes deterministic. Although organisms have been reported to behave in ways that reduce the uncertainty of predictions (Parr et al., 2022), they are nevertheless forced to make decisions under conditions of uncertainty. Hence, we can anticipate that WFL under the uncertain optimality may also be found in the biological relationship between TD errors and dopamine in organisms.

Through simulations and real-robot experiments, we also confirm that the RL algorithm incorporating the derived WFL can effectively learn optimal policies properly and exert special effects on learning processes and outcomes. In particular, the proposed RL algorithm acquires tasks, and the WFL added in the right balance maximizes rewards eventually while suppressing punishments during learning. In addition, the capability to accelerate learning from a small reward phase allows the robot to efficiently learn a valve-turning task (Ahn et al., 2020) on a real robot, decreasing the error from the target stably. Thus, WFL is useful in RL, raising expectations that organisms have the same (or similar) utilities.

## 2 Preliminaries

### 2.1 Reinforcement learning

In RL, an agent aims to optimize a learnable policy so that the accumulation of future rewards from an unknown environment (so-called return) is maximized (Sutton and Barto, 2018) under a Markov decision process (MDP). In other words, an environment with a task to be solved is (implicitly) defined as the tuple $\left(S,A,R,p_{0},p_{e}\right)$ . Here, $S\subset R^{\left|S\right|}$ and $A\subset R^{\left|A\right|}$ denote the state and action spaces, respectively, with the |S| -dimensional state s and the |A| -dimensional action a . R⊆R is the subset on which rewards exist, and the specific values (and even existences) of its upper and lower boundaries $R\subseteq \left(\underline{R},\bar{R}\right)$ are usually unknown. $p_{0}:S\mapsto R_{+}$ denotes the probability for sampling the initial state of each trajectory, and $p_{e}:S\times A\times S\mapsto R_{+}$ is known as the state transition probability (or dynamics).

With such a definition, the agent repeatedly interacts with the environment at the current state s according to the action a determined by its policy $\pi :S\times A\mapsto R_{+}$ with its learnable parameters ϕ , resulting in the next state $s^{\prime }$ and the corresponding reward r , which is computed using the reward function r:S×A↦R . As a result, the agent obtains the return $R_{t}$ from the time step t as presented in Equation 1:

$$R_{t}=\left(1-\gamma \right)\overset{\infty }{\underset{k=0}{\sum }}\gamma^{k}r_{t+k},$$ (1)

where γ∈[0,1) denotes the discount factor. Note that 1−γ is multiplied for normalization to match the implementation used in this study, although the definition without it is common.

The optimal policy π* is defined for this, as shown in Equation 2:

$$\pi^{*}\left(\cdot |s\right)=\arg\underset{\pi }{\max}E_{p_{\tau }}\left[R_{t}|s_{t}=s\right],$$ (2)

where $p_{\tau }$ denotes the probability for the trajectory, defined as the joint probability of $p_{e}$ and π from t to ∞ . ϕ is optimized to represent π* for any state.

As a remark, the maximization target is modeled as the (state) value function V:S↦R with its learnable parameters θ . When $a_{t}=a$ is also given as the additional condition for computing the above expectation as $E_{p_{\tau }}\left[R_{t}|s_{t}=s,a_{t}=a\right]$ , the action value function Q:S×A↦R is defined for the agent’s policy. Here, Q(s,a) can be approximated by $r+\gamma V\left(s^{\prime }\right)$ by following the recursive definition of return (i.e., Bellman equation), and the difference between it and V(s) is defined as the TD error, $\delta ≔r+\gamma V\left(s^{\prime }\right)-V\left(s\right)$ , which should be minimized as much as possible by optimizing θ for any state. In addition, δ can be utilized for updating ϕ so that π is more likely to generate actions that make δ more positive (i.e., larger return than expected).

### 2.2 Update rule derived from control as inference

To interpret the above optimal control problem as a type of inference problem, *control as inference* introduces the stochastic variable for the trajectory’s optimality O={0,1} (Levine, 2018). As it is relevant to the return, its conditional probability is defined as shown in Equation 3:

$$\begin{array}{cc} p\left(O=1|s\right) & =e^{\beta \left(V\left(s\right)-\bar{R}\right)}≕p_{V}\\ p\left(O=1|s,a\right) & =e^{\beta \left(Q\left(s,a\right)-\bar{R}\right)}≕p_{Q}, \end{array}$$ (3)

where $\beta \in R_{+}$ denotes the inverse temperature parameter. Note that p(O=0) can also be given as 1−p(O=1) since O is binary. From this definition, O can be explained so that it is more likely to be 1 if the value is higher. When β is small, optimality is ambiguous, and as β increases, optimality becomes deterministic.

With the probability of optimality, the optimal and non-optimal policies are inferred according to Bayes theorem. In particular, with the baseline policy b(a∣s) for sampling actions, π(a∣s,O) is obtained, as presented in Equation 4:

$$\begin{array}{ll} & \pi \left(a|s,O\right)=\frac{p\left(O|s,a\right)b\left(a|s\right)}{p\left(O|s\right)}\\ & =\left\{\begin{array}{cc} \frac{e^{\beta \left(Q\left(s,a\right)-\bar{R}\right)}}{e^{\beta \left(V\left(s\right)-\bar{R}\right)}}b\left(a|s\right)\; & O=1\\ \frac{1-e^{\beta \left(Q\left(s,a\right)-\bar{R}\right)}}{1-e^{\beta \left(V\left(s\right)-\bar{R}\right)}}b\left(a|s\right)\; & O=0. \end{array}\right. \end{array}$$ (4)

Based on this definition, a previous study (Kobayashi, 2022b) considered the minimization problem presented in Equation 5 for optimizing θ , the parameters of the value function V .

$$\underset{\theta }{\min}E_{p_{e},b}\left[KL\left(p\left(O|s\right)|p\left(O|s,a\right)\right)\right],$$ (5)

where $KL\left(p_{1}|p_{2}\right)=E_{x∼p_{1}}\left[\ln p_{1}\left(x\right)-\ln p_{2}\left(x\right)\right]$ is Kullback–Leibler (KL) divergence. More specifically, as the target probability is on the right side, this can be regarded as “reverse” KL divergence. Since p(O∣s,a) has more information than p(O∣s) , this minimization problem makes p(O∣s) more informative to represent the optimality. To solve this problem, its gradient with respect to θ , $g_{\theta }$ , is derived as shown in Equation 6:

$$\begin{array}{ll} g_{\theta } & =E_{p_{e},b}\left[\nabla_{\theta }p_{V}\ln\frac{p_{V}}{p_{Q}}-\nabla_{\theta }p_{V}\ln\frac{1-p_{V}}{1-p_{Q}}+p_{V}\nabla_{\theta }\ln p_{V}+\left(1-p_{V}\right)\nabla_{\theta }\ln\left(1-p_{V}\right)\right]\\ & =E_{p_{e},b}\left[-\nabla_{\theta }V\left(s\right)\beta p_{V}\left\{\beta \left(Q\left(s,a\right)-V\left(s\right)\right)+\ln\frac{1-p_{V}}{1-p_{Q}}\right\}\right]\\ & \propto E_{p_{e},b}\left[-\nabla_{\theta }V\left(s\right)\left\{\left(1-\lambda_{\beta }\right)\left(Q\left(s,a\right)-V\left(s\right)\right)+\lambda_{\beta }\ln\frac{1-p_{V}}{1-p_{Q}}\right\}\right], \end{array}$$ (6)

where $\lambda_{\beta }≔{\left(1+\beta \right)}^{-1}\in \left(0,1\right)$ . The last proportion is obtained by dividing the raw gradient by $\beta \left(1+\beta \right)p_{V}$ . Since β is constant, it can be absorbed into the learning rate, but $p_{V}$ appears to introduce a bias in the convergence point, as noted in the previous study (Kobayashi, 2022b). However, we found that the Fisher information for p(O|V) is given as $\beta^{2}p_{V}{\left(1-p_{V}\right)}^{-1}$ , and dividing the raw gradient by $p_{V}$ can be interpreted as the sum of the raw and natural gradients (details are in Appendix 1), which is expected to converge to the same destination without bias (Landro et al., 2020).

As a practical problem, $p_{V}$ and $p_{Q}$ cannot be numerically computed since they include the unknown $\bar{R}$ , the upper bound of the reward and return. The previous study approximates the above gradient by assuming $\lambda_{\beta }\to 0$ (i.e., β→∞ ), resulting in standard TD learning (by assuming $Q\left(s,a\right)≃r+\gamma V\left(s^{\prime }\right)$ ).

In addition to the value function, the policy π (more precisely, its parameter ϕ ) is also optimized through the following minimization problem, as presented in Equation 7, with the reverse KL divergences.

$$\begin{array}{ll} & \underset{\phi }{\min}E_{p_{e}}\left[KL\left(\pi \left(a|s\right)|\pi \left(a|s,O=1\right)\right)-KL\left(\pi \left(a|s\right)|\pi \left(a|s,O=0\right)\right)\right]\\ & \;=\underset{\phi }{\min}E_{p_{e},\pi }\left[\ln\frac{\pi \left(a|s,O=0\right)}{\pi \left(a|s,O=1\right)}\right]. \end{array}$$ (7)

In particular, the policy tries to be close to the optimal policy, while being far away from the non-optimal policy. The gradient with respect to ϕ , $g_{\phi }$ , is also derived analytically, as shown in Equation 8:

$$\begin{array}{ll} g_{\phi } & =E_{p_{e},\pi }\left[\frac{\nabla_{\phi }\pi \left(a|s\right)}{\pi \left(a|s\right)}\ln\frac{\pi \left(a|s,O=0\right)}{\pi \left(a|s,O=1\right)}\right]\\ & =E_{p_{e},\pi }\left[-\nabla_{\phi }\ln\pi \left(a|s\right)\left\{\beta \left(Q\left(s,a\right)-V\left(s\right)\right)+\ln\frac{1-p_{V}}{1-p_{Q}}\right\}\right]\\ & \propto E_{p_{e},b}\left[-\frac{\pi \left(a|s\right)}{b\left(a|s\right)}\nabla_{\phi }\ln\pi \left(a|s\right)\left\{\left(1-\lambda_{\beta }\right)\left(Q\left(s,a\right)-V\left(s\right)\right)+\lambda_{\beta }\ln\frac{1-p_{V}}{1-p_{Q}}\right\}\right], \end{array}$$ (8)

where the last proportion is given by dividing the raw gradient by (1+β) . In addition, at the final step, the importance sampling replaces π in the expectation operation with the baseline policy b . Along with the value function, the approximation of $\lambda_{\beta }\to 0$ makes this gradient computable, resulting in the standard policy gradient in actor-critic algorithms.

## 3 Weber–Fechner law in TD learning

### 3.1 Numerical analysis with an explicit upper bound

The gradients to optimize the value and policy functions are derived in Equations 6, 8, respectively. However, as the upper bound of the reward function $\bar{R}$ is unknown and $p_{V}$ and $p_{Q}$ cannot be calculated numerically, it was necessary to exclude the uncomputable term by setting $\lambda_{\beta }\to 0$ . As a result, the previous study (Kobayashi, 2022b) found the conventional update rule, where the gradients are weighted by $Q\left(s,a\right)-V\left(s\right)≃r+\gamma V\left(s^{\prime }\right)-V\left(s\right)=\delta$ (i.e., the TD error). On the other hand, if the nonlinear term excluded (i.e., $\delta_{\text{ln}}≔\ln\left(1-p_{V}\right)-\ln\left(1-p_{Q}\right)$ ) is computable, it is interesting how it affects the gradients, and this analysis is the main focus of this study.

**FIGURE 1:**
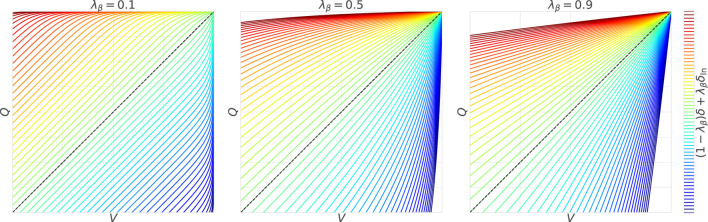
Effects of the nonlinear term $\delta_{\text{ln}}$ : when δ is dominant, the degrees of updates depicted by the contour lines are mostly equally spaced in parallel to the line of V=Q ; when the influence of $\delta_{\text{ln}}$ increases, the contour lines radiate out from the upper bound $\bar{R}$ .

Therefore, we assume that $\bar{R}$ is known at once in this section. With this assumption, the gradient including $\delta_{\text{ln}}$ is analyzed. First, we numerically visualize the gradient according to $\lambda_{\beta }\in \left(0,1\right)$ and estimate the role of $\delta_{\text{ln}}$ , which has a stronger influence when $\lambda_{\beta }$ increases (i.e., β decreases). For this purpose, $\left(1-\lambda_{\beta }\right)\delta +\lambda_{\beta }\delta_{\text{ln}}$ (i.e., the degree of updates) for $\lambda_{\beta }=\left\{0.1,0.5,0.9\right\}$ in the case R=(−1,1) is illustrated in Figure 1.

First, at $\lambda_{\beta }=0.1$ , the contour lines representing the degree of updates are spaced equally and parallel to the line of V=Q . This is mainly because δ is dominant; that is, the degree of updates is linearly proportional to the TD error. Note that the behavior is slightly different for $V,Q≃\bar{R}$ because $\delta_{\text{ln}}$ remains. The remaining $\delta_{\text{ln}}$ , however, easily converges to 0 since the large β ( =9 in this case) causes $p_{V}$ and $p_{Q}$ converge to 0, even with only a small difference between V,Q and $\bar{R}$ (i.e., optimality is deterministic).

On the other hand, when $\lambda_{\beta }=0.9$ , $\delta_{\text{ln}}$ is dominant, causing the contour lines to extend radially from $\bar{R}$ . In other words, when the value is close to $\bar{R}$ , the update is significantly activated even with a small TD error, whereas when the value is far from $\bar{R}$ , only a large TD error can sufficiently drive the update. Unlike the case with $\lambda_{\beta }=0.1$ , $\delta_{\text{ln}}$ has a strong effect even when the value is far from $\bar{R}$ because $p_{V}$ and $p_{Q}$ with the small β ( =1/9 in this case) are changed at approximately 1/2 without converging to 0 (i.e., optimality is uncertain).

Finally, $\lambda_{\beta }=0.5$ yields an intermediate behavior between the above two characteristics. In particular, when the value is somewhat close to $\bar{R}$ , the radial spread from $\bar{R}$ is observed due to the influence of $\delta_{\text{ln}}$ , and when it falls below a certain level, δ dominates, and it switches to parallel contour lines. However, it should be noted that this trend depends on the range of R , so it is not always true for $\lambda_{\beta }=\left\{0.1,0.5,0.9\right\}$ .

### 3.2 Mathematical analysis using the Taylor expansion

We further analyze the characteristics of $\delta_{\text{ln}}$ found in the above numerical results. Since these characteristics become apparent when V and Q are close to $\bar{R}$ , we apply a Taylor expansion to $p_{V}$ and $p_{Q}$ around $\bar{R}$ , as presented in Equations 9, 10:

$$p_{V}=\overset{\infty }{\underset{n=0}{\sum }}\frac{\beta^{n}{\left(V-\bar{R}\right)}^{n}}{n!}≃1+\beta \left(V-\bar{R}\right),$$ (9)

$$p_{Q}=\overset{\infty }{\underset{n=0}{\sum }}\frac{\beta^{n}{\left(Q-\bar{R}\right)}^{n}}{n!}≃1+\beta \left(Q-\bar{R}\right).$$ (10)

Accordingly, $\delta_{\text{ln}}$ is presented in Equation 11, [Disp-formula e10]:

$$\delta_{\text{ln}}=\ln\frac{1-p_{V}}{1-p_{Q}}≃-\ln\frac{\bar{R}-Q}{\bar{R}-V}.$$ (11)

At this point, let us interpret $\bar{R}-V$ as the baseline stimulus strength, $\bar{R}-Q$ as the stimulus strength after the change (or −δ in $\bar{R}-Q≃\bar{R}-V-\delta$ as the change in stimulus strength), and $-\delta_{\text{ln}}$ as the intensity of perception. If this is the case, these are subject to the WFL. In other words, how strongly the stimulus change −δ is perceived (i.e., $-\delta_{\text{ln}}$ ) depends on the baseline of the stimulus strength $\bar{R}-V$ : the smaller $\bar{R}-V$ is, the more acute the sensation becomes, and *vice versa*. This is exactly the characteristic found in the right side of Figure 1, indicating that the approximation by the Taylor expansion is valid.

We then conclude the analysis that WFL is hidden even in the update rule of RL derived from control as inference. WFL has also been found in areas closely related to brain functions such as neuron firing patterns (Scheler, 2017) and cognition (Portugal and Svaiter, 2011). Recently, it has been shown that the time steps in RL can theoretically be a nonlinear log scale (i.e., WFL), leading to adaptive temporal discounting (Maini and Tiganj, 2025). Therefore, it is not implausible to find it in RL, which is also attracting attention as a biological decision-making model (Dayan and Balleine, 2002; Doya, 2021). This hypothesis would be supported by the fact that WFL is activated when optimality is uncertain, which is consistent with the conditions faced by organisms (Parr et al., 2022).

Furthermore, its applicability to learning and practical engineering value should be verified through numerical experiments, with the exception that WFL represents a useful characteristic in RL and has been evolutionary preserved in organisms. However, the above analysis was performed under the assumption that R is known, which is contrary to the general problem statement for RL. In the next section, therefore, we propose a practical implementation that enables the computation of $\delta_{\text{ln}}$ even when R is unknown in a biologically plausible manner, followed by an experimental verification of the benefits of WFL in RL.

## 4 Practical implementation

### 4.1 Introduction of the reward–punishment framework

First, we address the unknown R without giving prior knowledge of the problem to be solved. The requirements are i) the boundary of R to define the optimality and ii) the range of R to determine β (or $\lambda_{\beta }$ ) for which WFL is valid. A naive solution would be to empirically estimate the boundary $\left(\underline{R},\bar{R}\right)$ . The estimation of the range $\left(\bar{R}-\underline{R}\right)$ does not need to be rigorous, so learning can proceed stably if it is updated slowly. However, for $p_{V}$ and $p_{Q}$ to consistently satisfy the definition of probability, $\bar{R}$ may need to change frequently, which could cause instability in learning. Moreover, the empirically estimated $\bar{R}$ is likely to be underestimated relative to its true value, thereby preventing the full utility of WFL from being realized.

**FIGURE 2:**
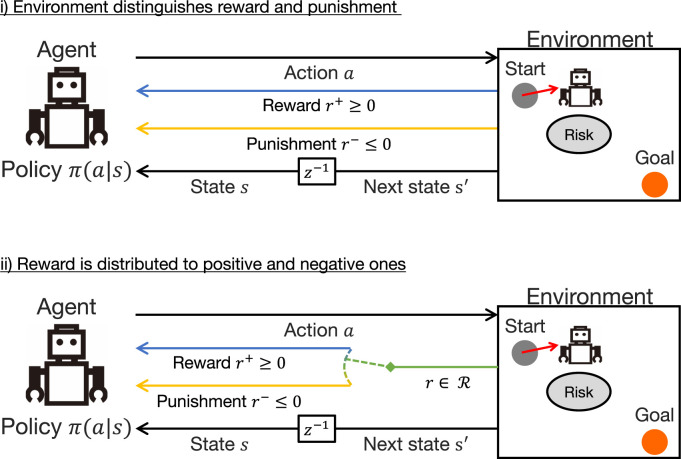
Reward–punishment framework: in the upper case, both rewards and punishments are directly given from the environment; in the bottom case, scalar rewards in a subset of real space are treated by distinguishing between positive and negative ones as rewards and punishments, respectively.

Therefore, in this study, we introduce a more reliable solution, the *reward–punishment framework* (Kobayashi et al., 2019; Wang et al., 2021). Although rewards are generally defined as scalar values that can be either positive or negative, this is a way to separate positive rewards $r^{+}\in R_{+}\subseteq R_{+}$ and negative rewards, i.e., punishments, $r^{-}\in R_{-}\subseteq R_{-}$ . This can be applied to any RL problem, either i) by having the environment output $r^{+,-}$ as in the experiments of this study (see the top of Figure 2) or ii) by distributing r∈R from the environment as $r^{+}=\max\left(r,0\right)$ and $r^{-}=\min\left(r,0\right)$ (see the bottom of Figure 2).

The reward–punishment framework learns the value and policy functions for $r^{+,-}$ , respectively. In particular, the returns and the value functions for $r^{+,-}$ are first defined as shown in Equation 12, [Disp-formula e10]:

$$\begin{array}{cc} R_{t}^{+,-} & =\left(1-\gamma \right)\overset{\infty }{\underset{k=0}{\sum }}\gamma^{k}r_{t+k}^{+,-}\\ V^{+,-}\left(s\right) & =E_{p_{\tau }}\left[R_{t}^{+,-}|s_{t}=s\right]\\ Q^{+,-}\left(s,a\right) & =E_{p_{\tau }}\left[R_{t}^{+,-}|s_{t}=s,a_{t}=a\right]. \end{array}$$ (12)

The policies $\pi^{+,-}$ , which attempt to maximize them separately, are also introduced.

Here, since only one action can be passed to the environment, even if the agent has two policies, it is necessary to synthesize them. Following the previous study (Wang et al., 2021), a mixture distribution with a mixing ratio based on the value function is designed, as shown in Equations 13, 14:

$$b\left(a|s\right)=w\pi^{+}\left(a|s\right)+\left(1-w\right)\pi^{-}\left(a|s\right),$$ (13)

$$w=\frac{e^{\beta_{w}V^{+}\left(s\right)}}{e^{\beta_{w}V^{+}\left(s\right)}+e^{-\beta_{w}V^{-}\left(s\right)}}.$$ (14)

With this design, however, only one of the policies might be activated and the other might be ignored if the difference in the scales of $r^{+,-}$ is large. To alleviate this issue, a policy regularization method, PPO-RPE (Kobayashi, 2023), for the density ratio $\pi^{+,-}/b$ with importance sampling [see Equation 8], is introduced in this study. As it yields $\pi^{+}≃\pi^{-}$ *via* b , the past mixture distributions transfer and share the acquired skills with each other. In addition, as PPO-RPE is a type of actor-critic algorithm, it can be applied not only to continuous but also to discrete action spaces.

In any case, within the reward–punishment framework, the upper bound of $r^{-}$ is given to be 0, making $\delta_{\text{ln}}$ computable. On the other hand, $r^{+}$ has only a lower bound of 0, so $\delta_{\text{ln}}$ for it remains uncomputable. In the next section, therefore, we derive $\delta_{\text{ln}}$ utilizing this lower bound.

The range of rewards, $\sigma^{+,-}$ , which is necessary for designing β where WFL is effectively manifested, can be estimated from the empirical $r^{+,-}$ . However, the assumption when deriving Equations 6, 8 (i.e., β is constant) is violated if $\sigma^{+,-}$ fluctuates too much. In addition, since the experienced scale of $r^{-}$ is likely to gradually decrease, the approach to record the maximum scale is not suitable for this case. From the above, $\sigma^{+,-}$ is estimated and used for the design of β as shown in Equation 15, [Disp-formula e8]:

$$\begin{array}{cc} \sigma_{\text{max}}^{+,-} & \leftarrow \max\left(\zeta \sigma_{\text{max}}^{+,-},|r^{+,-}|\right)\\ \sigma^{+,-} & \leftarrow \zeta \sigma^{+,-}+\left(1-\zeta \right)\sigma_{\text{max}}^{+,-}\\ \beta^{+,-} & =\frac{\beta_{0}}{\sigma^{+,-}}, \end{array}$$ (15)

where ζ∈(0,1) denotes the gradualness of adaptation (generally, ζ is close to 1) and $\beta_{0}\in R_{+}$ denotes the baseline. This design allows β to reflect the scale while limiting frequent fluctuations of β by using the recent maximum scale and updating to that value gradually. In addition, as this update rule does not use $V^{+,-}$ , it can avoid adverse effects due to the estimation uncertainty of $V^{+,-}$ .

### 4.2 Inversion of the definition of optimality using the lower bound

As mentioned above, although $r^{-}\leq 0$ can numerically compute Equations 6, 8 without any approximation, $r^{+}\geq 0$ cannot do so since it only has the lower bound. To solve this issue, we introduce a new method for deriving gradients with WFL, inspired by a previous study (Kobayashi, 2024b). In particular, the inversion of the definition of optimality in Equation 3 is considered the starting point.

$$\begin{array}{cc} p\left(O=0|s\right) & =e^{-\beta \left(V\left(s\right)-\underline{R}\right)}≕p_{V}\\ p\left(O=0|s,a\right) & =e^{-\beta \left(Q\left(s,a\right)-\underline{R}\right)}≕p_{Q}. \end{array}$$ (16)

In Equation 16, the lower bound $\underline{R}$ of R is utilized for satisfying the definition of probability. Note that the aliases $p_{V}$ and $p_{Q}$ are given for p(O=0) , unlike Equation 3.

As the previous study did not derive the gradients of Equations 5, 7 using Equation 16, their derivations are described below. First, $g_{\theta }$ for Equation 5 is derived as shown in Equation 17:

$$\begin{array}{ll} g_{\theta } & =E_{p_{e},b}\left[\nabla_{\theta }p_{V}\ln\frac{p_{V}}{p_{Q}}-\nabla_{\theta }p_{V}\ln\frac{1-p_{V}}{1-p_{Q}}+p_{V}\nabla_{\theta }\ln p_{V}+\left(1-p_{V}\right)\nabla_{\theta }\ln\left(1-p_{V}\right)\right]\\ & \propto E_{p_{e},b}\left[-\nabla_{\theta }V\left(s\right)\left\{\left(1-\lambda_{\beta }\right)\left(Q\left(s,a\right)-V\left(s\right)\right)-\lambda_{\beta }\ln\frac{1-p_{V}}{1-p_{Q}}\right\}\right]. \end{array}$$ (17)

Except for the different definitions of $p_{V}$ and $p_{Q}$ and the sign reversal of the second term, it is symmetric to Equation 6. Similarly, $g_{\phi }$ for Equation 7 is shown in Equation 18:

$$\begin{array}{ll} g_{\phi } & =E_{p_{e},\pi }\left[\frac{\nabla_{\phi }\pi \left(a|s\right)}{\pi \left(a|s\right)}\ln\frac{\pi \left(a|s,O=0\right)}{\pi \left(a|s,O=1\right)}\right]\\ & \propto E_{p_{e},b}\left[-\frac{\pi \left(a|s\right)}{b\left(a|s\right)}\nabla_{\phi }\ln\pi \left(a|s\right)\left\{\left(1-\lambda_{\beta }\right)\left(Q\left(s,a\right)-V\left(s\right)\right)-\lambda_{\beta }\ln\frac{1-p_{V}}{1-p_{Q}}\right\}\right], \end{array}$$ (18)

where $\pi \left(a|s,O=0\right)=p_{Q}p_{V}^{-1}b\left(a|s\right)$ and $\pi \left(a|s,O=1\right)=\left(1-p_{Q}\right){\left(1-p_{V}\right)}^{-1}b\left(a|s\right)$ are also redefined in this study. This gradient is also in the same format as Equation 17, except that the sign of the second term is reversed, with different definitions for $p_{V}$ and $p_{Q}$ . Note that the impact of redefining optimality can be confirmed when the minimization problem is designed with “forward” KL divergence, as analyzed in the previous study (Kobayashi, 2024b).

**FIGURE 3:**
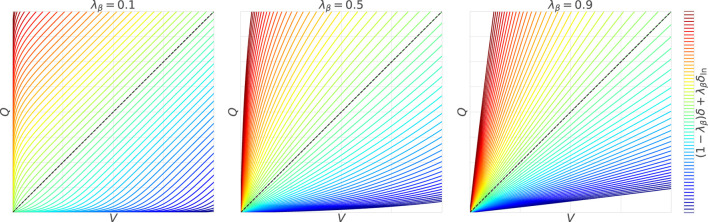
Weber–Fechner law using the lower bound: with the large $\lambda_{\beta }$ , the contour lines become narrower with V and Q closer to their lower bound $\underline{R}$ and wider with V and Q farther from $\underline{R}$ .

Both have the same degree of updates multiplied by the gradients, and the first term coincides with the TD error δ as in the original. The crucial second term appears to be different, but as depicted in Figure 3, the contour lines extend radially from $\underline{R}$ , as in the original. If the Taylor expansion around $\underline{R}$ is applied to $p_{V}$ and $p_{Q}$ , which are substituted into $\delta_{\text{ln}}≔-\ln\left(1-p_{V}\right)+\ln\left(1-p_{Q}\right)$ , the same WFL is confirmed. Thus, it is possible to compute the gradients with WFL even for $r^{+}$ , where only $\underline{R}=0$ is known.

### 4.3 Expected utilities

As described above, we proposed a novel algorithm including the terms with WFL, which had been excluded in the previous study (Kobayashi, 2022b) (and the standard RL algorithms) because they are computationally infeasible. Table 1 summarizes the correspondence between WFL and the update rule in the proposed algorithm. Note that since WFL is a law about the signal strength, the terms in punishments are converted for the punishment strength by reversing their signs.

**TABLE 1:** Correspondence between WFL and the proposed update rule.

	WFL	Update rule for $r^{+}$	Update rule for $r^{-}$
Formula	$p\propto \frac{S}{S_{0}}$	$\delta_{\text{ln}}\propto \ln\frac{Q}{V}$	$-\delta_{\text{ln}}\propto \ln\frac{-Q}{-V}$
Baseline of stimulus strength	$S_{0}$	V	−V
Stimulus strength after change	S	Q	−Q
Change in stimulus strength	$S-S_{0}$	Q−V≃δ	−(Q−V)≃−δ
Intensity of perception	p	$\delta_{\text{ln}}$	$-\delta_{\text{ln}}$

In this study, we summarize the basic utilities of WFL in this algorithm. First, for rewards $r^{+}$ , the updates of the value and policy functions are actively promoted at $V^{+}≃0$ , whereas the updates are relatively suppressed over a certain level, $V^{+}\gg 0$ . Conversely, for punishments $r^{-}$ , the updates are slow under a certain level $V^{-}\ll 0$ , but $V^{-}≃0$ is pursued eventually. It is known in gradient-based optimization that large gradients per update lead to a solution robust to small perturbations, while small gradients lead to one of the local solutions (Smith, 2018; Foret et al., 2021). Therefore, WFL in the proposed algorithm can also be interpreted as seeking an early local solution for $r^{+}$ and a stable global solution for $r^{-}$ . Note that, as shown in Figures 1, 3, the utilities of WFL can be suppressed by adjusting $\lambda_{\beta }$ (or β ) with the activation of the standard TD learning, which is not affected by the baseline stimulus strength (i.e., |V| ).

## 5 Numerical verification

### 5.1 Toy problem

First, we investigate the feasibility of learning the optimal policy under the proposed algorithm with WFL and the effects of WFL on the learning process and results. As a toy problem, *Pendulum-v0* implemented in OpenAI Gym is applied, while its reward function is redefined to fit the reward–punishment framework.

$$\begin{array}{cc} r^{+} & =1+\cos q\\ r^{-} & =-\left(0.1|\dot{q}|+0.001|\tau |\right)\left(1+\cos q\right), \end{array}$$ (19)

In Equation 19, q denotes the pendulum angle, $\dot{q}$ denotes its angular velocity, and τ denotes the torque applied to the pendulum (i.e., action). In particular, the agent gets high rewards if the pendulum is close to upright, while it is punished when the pendulum is not stopped. In addition, this punishment is stronger when the pendulum is close to upright. Since we know that *Pendulum-v0* is a standard and classic benchmark that eventually yields an optimal policy, it is useful for analyzing the changes in the learning process.

**FIGURE 4:**
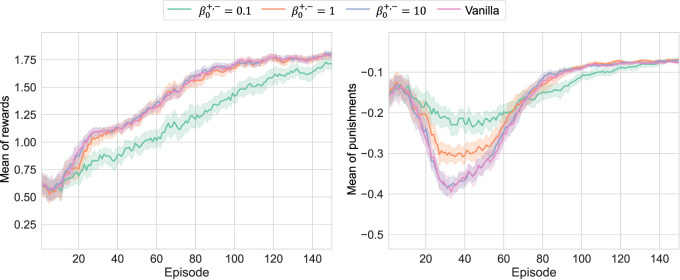
Learning results of *Pendulum-v0* with different $\beta_{0}^{+,-}$ : the upper and bottom curves depict the learning curves for episodic averages of $r^{+}$ and $r^{-}$ , respectively; when $\beta_{0}^{+,-}$ was small, the pursuit of $r^{+}$ became slower, and $r^{-}$ was preferentially suppressed to 0.

With $\beta_{0}^{+,-}=\left\{0.1,1,10,\infty \right\}$ ( ∞ indicates conventional TD learning), four respective models are trained 50 times with different random seeds in order to achieve the statistical learning results shown in Figure 4. Note that the learning conditions, including network architectures, are summarized in Appendix 2. As shown in the results, learning can proceed for any $\beta_{0}^{+,-}$ without collapse, indicating that RL is valid even if the term $\delta_{\text{ln}}$ with WFL is used. The increase in $\beta_{0}^{+,-}$ made the learning curves approach the conventional curves, as expected. On the other hand, when $\beta_{0}^{+,-}$ becomes smaller, unique behaviors were observed. In other words, for $\beta_{0}^{+,-}=0.1$ , optimization with respect to $r^{+}$ was delayed, while the temporary deterioration of $r^{-}$ was restricted. This can be attributed to the fact that optimization worked to reduce $r^{-}$ to 0 as much as possible while simultaneously maximizing $r^{+}$ to some extent. However, in the former case, it might be possible that the strong effort to reduce $r^{-}$ to 0 suppressed the exploration, causing a delay in the discovery of the optimal solution for $r^{+}$ . Note that $\beta_{0}^{+,-}=1$ achieved the similar learning curve of reward to the case of Vanilla due to the nonlinear effect of $\beta_{0}^{+,-}$ , but their confidence intervals were hardly overlapped at the early stages of learning.

**FIGURE 5:**
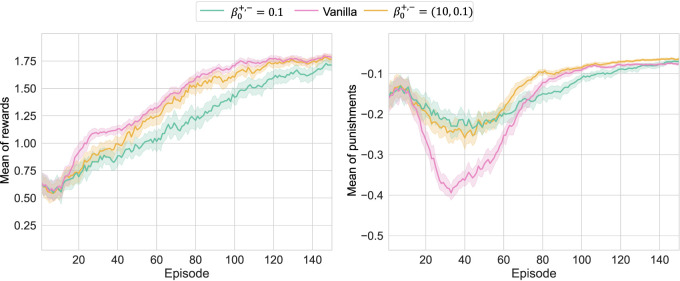
Learning results of *Pendulum-v0* with the asymmetric $\beta_{0}^{+,-}$ : because of small $\beta_{0}^{-}=0.1$ , the deterioration of $r^{-}$ was restricted; although the exploration was more or less limited, the large $\beta_{0}^{+}=10$ value enabled to maximize $r^{+}$ to the same level as the conventional method.

Then, to take the advantages of both, the results of setting $\beta_{0}^{+}=10$ and $\beta_{0}^{-}=0.1$ asymmetrically are depicted in Figure 5. Under this setting, WFL’s efforts to reduce $r^{-}$ to 0 remained and suppressed the temporary deterioration of $r^{-}$ , while $r^{+}$ was successfully optimized without much delay. In other words, the delay in learning about $r^{+}$ at $\beta_{0}^{+,-}=0.1$ can be attributed to the characteristics of WFL. Note that the delay in maximizing $r^{+}$ at approximately 20 episodes is considered an effect of the suppression of exploration. On the other hand, $r^{+}$ was maximized more efficiently, and $r^{-}$ was smaller in conjunction with it.

In any case, WFL’s utilities analyzed in this study were confirmed as expected in the numerical verification. In addition, as suggested in Figure 5, the optimization behaviors for $r^{+,-}$ can be adjusted by setting $\beta_{0}^{+,-}$ separately. However, we need to remark that the separation of $\beta_{0}^{+,-}$ does not mean that they function independently since $r^{+,-}$ necessarily depend on each other in the learning process, as in the exploration suppression described above.

### 5.2 Robotic task

**FIGURE 6:**
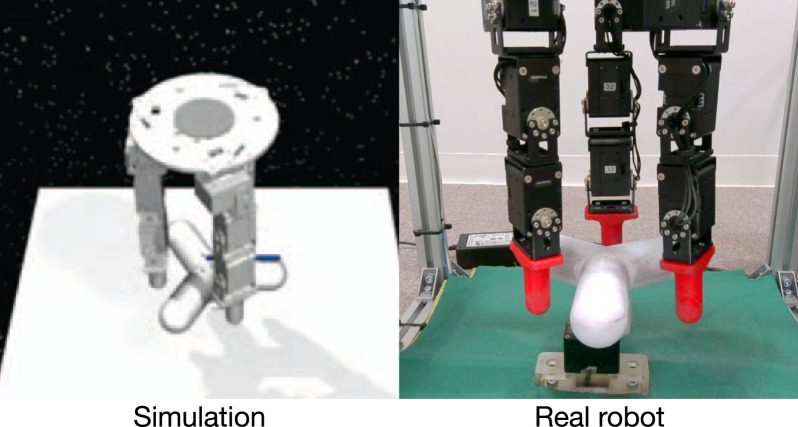
D’Claw task (Ahn et al., 2020): it is simulated on MuJoCo (Todorov et al., 2012).

The above numerical verification showed that the proposed method with WFL can optimize the policies with its expected learning characteristics. Based on this finding, we additionally demonstrate that the proposed method can be useful in more practical robotic tasks. This study focuses on the D’Claw task in ROBEL (Ahn et al., 2020), in which three 3-DOF robotic fingers manipulate a valve (see Figure 6). This benchmark is unique in that it includes not only a robotic simulation but also a real-robot version, which can automatically be initialized to restart episodes. Hence, this task is useful to verify that the proposed algorithm works in a real-world setting. Note that the code for this system is not the original code but a modified version in the literature (Yamanokuchi et al., 2022). Its state space consists of the angles and angular velocities of the finger joints and of the valve (in total, 22 dimensions), while the action space consists of nine dimensions of angular changes in the finger joints.

#### 5.2.1 Simulations

First, the simulations confirm that the behaviors when WFL is activated for $r^{+,-}$ individually are consistent with the toy problem. Since $\beta_{0}^{+,-}=0.1$ was too extreme and $\beta_{0}^{+,-}=1$ was insufficient for WFL effects, $\beta_{0}^{+,-}=0.5$ is adopted to activate WFL from here as *WFL-R/P*.

The reward function is defined as shown in Equation 20:

$$\begin{array}{cc} r^{+} & =q_{v}I_{q_{v}>0}\\ r^{-} & =-0.01\|q_{j}+\Delta q_{j}{\|}_{2}^{2}, \end{array}$$ (20)

where $q_{v}$ and $q_{j}$ denote the valve angle and the joint angles, respectively, and $\Delta q_{j}$ denotes the angular changes in joints (i.e., action). In particular, the goal is to turn the valve as much as possible while keeping the fingers in the initial posture to some extent. Note that the sign of $q_{v}$ reverses after one turn, so the actual goal is to stop just before one turn.

**FIGURE 7:**
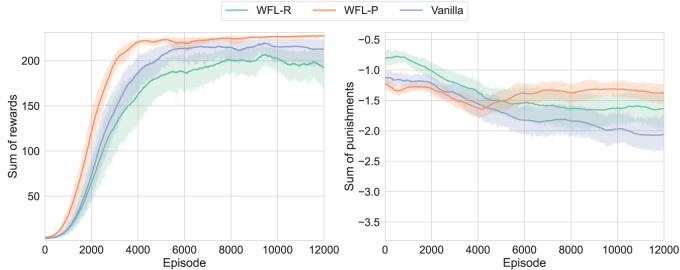
Learning results of D’Claw simulation: $\beta_{0}^{+,-}=\left(0.5,\infty \right),\left(\infty ,0.5\right)$ are labeled as *WFL-R* and *WFL-P*, respectively; the proposed method with WFL (especially, WFL-P) improved the maximization of rewards while reducing punishments.

The learning results of each condition with 20 different random seeds are shown in Figure 7. Here, *WFL-R* and *WFL-P* denote the asymmetric models with $\beta_{0}^{+,-}=\left(0.5,\infty \right),\left(\infty ,0.5\right)$ , respectively. Note that because the punishments were very small, unlike in the toy problem, we plotted the sum of rewards/punishments per episode rather than their mean. First, the pursuit of $r^{+}$ was slowed down in *WFL-R* with WFL for $r^{+}$ . As its side effect, $r^{-}$ always outperformed the conventional method due to the reduced robot motion. On the other hand, *WFL-P* with WFL for $r^{-}$ showed improvement after approximately 4000 episodes, reflecting the pursuit of reducing punishments and finally achieving the best performance. In addition, probably because the range of motion of each finger joint was maintained as its side effect, the speed of improvement in $r^{+}$ was significantly increased compared to others. Thus, it was suggested that the appropriate addition of WFL, including its side effects, can improve learning performance in practical robotic tasks.

#### 5.2.2 Real-robot experiments

Next, we demonstrate how WFL works in learning on the real world. For simplicity, WFLs for $r^{+,-}$ are both activated simultaneously and compared with the conventional method.

Since the real-robot valve angle has a different domain from the simulation angle and the angle jumps to π→−π in a half turn, the reward function is modified accordingly, as shown in Equation 21:

$$\begin{array}{cc} r^{+} & =|q_{v}|I_{q_{v}>0\vee q_{v}<-\frac{3}{4}\pi }\\ r^{-} & =-0.01\|q_{j}+\Delta q_{j}{\|}_{2}^{2}. \end{array}$$ (21)

In other words, the goal is to stop the valve half a turn while allowing some overshoot. Note that, as the other differences from the above simulations, the ranges of motion and actions (i.e., the exploration capability) are restricted to avoid hardware malfunction.

**FIGURE 8:**
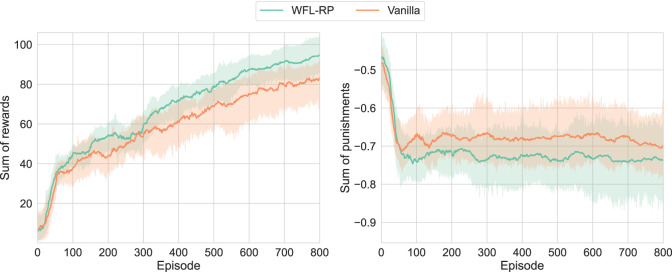
Learning results of real-world D’Claw: $\beta_{0}^{+,-}=\left(0.5,0.5\right)$ is labeled as *WFL-RP*; accelerating the increase in a small reward was confirmed, although the increase in punishment by the side effect of the behavior for rotating the valve was not suppressed.

First, the learning results with five trials are shown in Figure 8. Note that it was difficult to verify the asymmetric $\beta_{0}^{+,-}$ due to the cost of the real-robot experiments, even with automatic episode initialization. Therefore, the robustness of the proposed method to hyperparameters is demonstrated by adopting the symmetric *WFL-RP* as the proposed method under the assumption that there is limited prior knowledge (i.e., *WFL-P* was better in the simulations).

It was confirmed in $r^{+}$ that the proposed method always outperformed the conventional method. This is probably because the proposed method preferentially learned a small number of motion samples that rotated the valve forward, which were rarely obtained by chance with the limited exploration capability. Instead, $r^{-}$ of the proposed method was slightly lower than that of the conventional method, probably because the side effect of the behavior to rotate the valve was larger than the behavior to maintain $r^{-}$ at 0. Another possibility that should be noted is that the regularization of $\pi^{+}≃\pi^{-}$ was added, but the large difference in scale between $r^{+}$ and $r^{-}$ may have prevented it from functioning satisfactorily, and the policy to pursue $r^{+}$ may have been prioritized.

**FIGURE 9:**
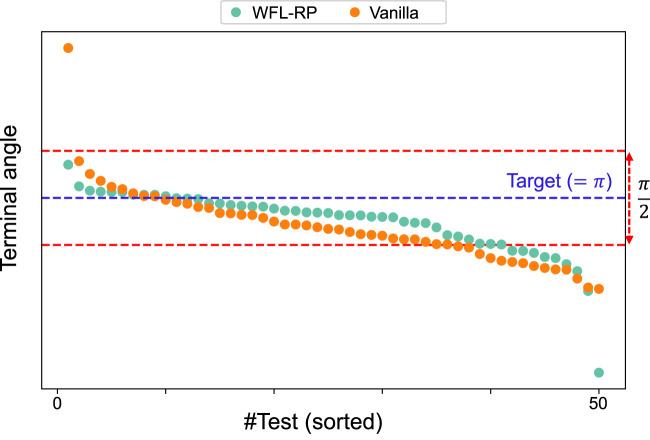
Terminal valve angles with the post-learning policies: 10 episodes were performed in each of the 5 models, and the (sorted) valve angles at the end of the episodes were plotted; *WFL-RP* tended to be closer to the target angle than that in the conventional method, and it also outperformed in the success rate where the error was within π/4 .

Next, task accomplishment, which cannot be evaluated from $r^{+,-}$ alone, is evaluated using the terminal valve angle with the post-learning policies. The five post-learning policies for each condition tested 10 episodes, as shown in Figure 9. As expected from the learning curve of $r^{+}$ , the proposed method produced more results closer to the target angle, θ=π . Moreover, when the range of ±π/4 from the target angle is considered the success, the proposed method showed 41/50 (i.e., 82% ), whereas the conventional method showed 35/50 (i.e., 70% ).

## 6 Discussion and conclusion

### 6.1 Discussion

As shown above, although WFL in TD learning was confirmed in this study can be shown to produce more desirable learning processes and outcomes, whether WFL is more sensitive to reward design than conventional TD learning is remains an open question. For example, an inappropriate design may cause a conflict between the reward and punishment policies, potentially preventing them from achieving their respective objectives. Basically, objectives given as punishments $r^{-}$ should have a high priority for achievement, and those with rewards $r^{+}$ should be regarded as value-added. However, prioritization among multiple objectives is often given as weights, which may lead to overlapping roles among multiple parameters and make it difficult to understand. The complexity is further increased by the fact that the impact of WFL can be adjusted by $\lambda_{\beta }$ (or β ).

Therefore, the need to assign such priorities to RL users and/or task designers may pose an obstacle to real-world applications. To alleviate this issue, further research on the design theory of reward functions suitable for this algorithm and/or the automation of assignment to $r^{+,-}$ (and tuning of hyperparameters) based on user preferences would increase the practical value of this algorithm. Recently, it might be a good idea to specify the context in LLM-based reward design (Ma et al., 2024) so that the necessary factors are set as $r^{-}$ and those to be desired are $r^{+}$ , while avoiding the conflict between them.

On the other hand, WFL, found in TD learning, originally explains the relationship between stimuli and perception in organisms, but it has not been discovered in brain activities related to TD learning. Considering that RL is also used as decision-making models for organisms and that the relationship between TD errors and brain activities has actually been verified, it is possible that WFL in TD learning may be latent in our brain activities.

Therefore, it would be important to verify the existence or absence of WFL using this algorithm for the analysis of brain-activity data. Moreover, the feedback from the findings may be able to elaborate on our algorithm: for example, the hyperparameters in the algorithm might be tuned by representing the brain-activity data. When conducting this investigation, it may be possible to derive a more sophisticated model if the WFL in the time direction derived by Maini and Tiganj (2025) can also be considered in addition to the proposed algorithm with the WFL in TD errors. Alternatively, brain-activity data may provide suggestions for new modeling of the heuristic update rule of $\beta^{+,-}$ .

### 6.2 Conclusion

In this study, we revealed a novel nonlinearity in TD learning, WFL, which explains the relationship between stimuli and perception of organisms in the update rule of the value and policy functions in RL. Without loss of generality, it was implemented as a novel biologically plausible RL algorithm on the reward–punishment framework. We showed that the proposed method can be expected to explore a local solution to maximize rewards as early as possible while gradually aiming for a global solution to minimize punishments. Numerical verification indicated that the proposed method does not cause RL to collapse and retains the characteristics of WFL. The proposed method was also useful for robot control, and it outperformed the conventional method in the valve-turning task using D’Claw.

After addressing the limitations identified in this study, it would be valuable to test its generalizability (e.g., its capability to learn tasks with sparse rewards and/or discrete action spaces).

## Data Availability

The raw data supporting the conclusions of this article will be made available by the authors, without undue reservation.

## Appendix A

### 1 Natural gradient for θ

The Fisher information for p(O|V) , I(V) , is derived as follows:

$$\begin{array}{ll} I\left(V\right) & =E_{p\left(O|V\right)}\left[{\left(\frac{\partial }{\partial V}\ln p\left(O|V\right)\right)}^{2}\right]=p\left(O=1|V\right){\left(\frac{\partial }{\partial V}\ln p\left(O=1|V\right)\right)}^{2}\\ & \;+p\left(O=0|V\right){\left(\frac{\partial }{\partial V}\ln p\left(O=0|V\right)\right)}^{2}=\beta^{2}p_{V}+\left(1-p_{V}\right){\left(\frac{-\beta p_{V}}{1-p_{V}}\right)}^{2}\\ & =\beta^{2}p_{V}\left(1+\frac{p_{V}}{1-p_{V}}\right)=\beta^{2}\frac{p_{V}}{1-p_{V}} \end{array}$$
(22)

where $p_{V}=p\left(O=1|V\right)=e^{\beta \left(V-\bar{R}\right)}$ .

The natural gradient is obtained by dividing the raw gradient by the Fisher information (Amari, 1998). As the raw gradient $g^{\text{raw}}$ is represented as $-\left\{\left(1-\lambda_{\beta }\right)\delta +\lambda_{\beta }\delta_{\text{ln}}\right\}\beta \left(1+\beta \right)p_{V}\nabla_{\theta }V$ , its natural gradient $g^{\text{nat}}$ can be given as $-\left\{\left(1-\lambda_{\beta }\right)\delta +\lambda_{\beta }\delta_{\text{ln}}\right\}\beta^{-1}\left(1+\beta \right)\left(1-p_{V}\right)\nabla_{\theta }V$ . By removing the constant coefficients related to β , their summation can be derived as $-\left\{\left(1-\lambda_{\beta }\right)\delta +\lambda_{\beta }\delta_{\text{ln}}\right\}\nabla_{\theta }V$ .

### 2 Learning conditions

The value and policy functions for $r^{+,-}$ are independently approximated by a common network structure. The structure has two fully connected layers as hidden layers, with 100 neurons for each, and ReLU function is employed as its activation function. AdaTerm (Ilboudo et al., 2023) is employed to optimize the network parameters, and its learning rate is set to $1\times 10^{-3}$ for the toy problem and $5\times 10^{-4}$ for the robotic task. The sample efficiency is improved with experience replay, although its buffer size is small (i.e. $1\times 10^{4}$ ) to emphasize on-policyness. Half of the stored experiences are randomly replayed at the end of each episode with 32 the batch size. As tricks to stabilize learning, we introduce PPO-RPE (Kobayashi, 2023) introduced in the main text, target networks with CAT-soft update (Kobayashi, 2024a), and regularization to make the functions smooth by L2C2 (Kobayashi, 2022a). All of these remain the default hyperparameters. Other hyperparameters are γ=0.99 for the discount rate and ζ=0.999 for the empirical TD error scale estimation in Equation 15.
